# Susceptibility to SARS-CoV-2 Infection and Immune Responses to COVID-19 Vaccination Among Recipients of Solid Organ Transplants

**DOI:** 10.1093/infdis/jiad152

**Published:** 2023-08-04

**Authors:** Vijay Subramanian

**Affiliations:** Transplant Institute, Tampa General Hospital and University of South Florida Morsani School of Medicine, Tampa, Florida, USA

**Keywords:** COVID-19, immunocompromised, mRNA vaccine, SARS-CoV-2, solid organ transplant

## Abstract

Solid organ transplant recipients (SOTRs) are at high risk for infections including SARS-CoV-2, primarily due to use of immunosuppressive therapies that prevent organ rejection. Furthermore, these immunosuppressants are typically associated with suboptimal responses to vaccination. While COVID-19 vaccines have reduced the risk of COVID-19–related morbidity and mortality in SOTRs, breakthrough infection rates and death remain higher in this population compared with immunocompetent individuals. Approaches to enhancing response in SOTRs, such as through administration of additional doses and heterologous vaccination, have resulted in increased seroresponse and antibody levels. In this article, safety and immunogenicity of mRNA COVID-19 vaccines in SOTRs are explored by dose. Key considerations for clinical practice and the current vaccine recommendations for SOTRs are discussed within the context of the dynamic COVID-19 vaccination guideline landscape. A thorough understanding of these topics is essential for determining public health and vaccination strategies to help protect immunocompromised populations, including SOTRs.

## SOLID ORGAN TRANSPLANT RECIPIENTS FACE A HIGH RISK FOR COVID-19

Solid organ transplantation is a well-established therapeutic option that reduces mortality and improves quality of life in patients with terminal organ failure and certain types of liver cancer. The Global Observatory on Donation and Transplantation reported 144 302 solid organ transplants (SOTs) in 2021 [1]. In the United States, 42 888 SOTs were performed in 2022, including kidney (n = 25 499), liver (n = 9528), heart (n = 4111), lung (n = 2692), pancreas (n = 108), and intestine (n = 82) transplants [2].

Increasingly effective posttransplant immunosuppressive therapies that prevent organ rejection (eg, corticosteroids, calcineurin inhibitors [cyclosporine and tacrolimus], mTOR inhibitors [everolimus], azathioprine, and mycophenolate mofetil [MMF]) have advanced graft and patient survival rates, yet continue to place transplant recipients at high risk for infections [3–7]. The incidence of infectious pathogens differs by organ type; however, viral, bacterial, and fungal infections are a major cause of morbidity and mortality among all SOT recipients (SOTRs) [3, 4, 6, 8–10]. Immunosuppressive therapies are also generally associated with poor immune responses to vaccination in SOTRs [5, 8, 9]. In particular, MMF is known to completely disturb responses to a number of different vaccines [7]. For example, seroconversion rates following influenza vaccination among adult and pediatric SOTRs were low and not notably enhanced by certain alternative strategies, such as intradermal or adjuvant-containing vaccines (5%–64%). Nevertheless, some studies suggest that high-dose or booster influenza vaccination strategies may slightly improve seroconversion rates among SOTRs [9, 11].

Unsurprisingly, SOTRs also have an elevated risk for SARS-CoV-2 infection and poor COVID-19–related outcomes and death [12–14]. Early reports indicated that compared with nontransplant patients, hospitalized SOTRs had substantially higher rates of severe COVID-19 disease (6% vs 39%) and mortality (1%–4% vs 24%) [12, 15–17]. The risk of adverse outcomes following COVID-19 diagnosis varies significantly by organ type; heart and kidney transplant recipients are at highest risk. Furthermore, compared with immunocompetent individuals, SOTRs are at significantly higher risk of acute kidney injury and major adverse cardiac events arising from COVID-19–related immune dysregulation [12].

As the COVID-19 pandemic progressed, vaccines were developed and authorized for emergency use. Despite the exclusion of SOTRs from initial phase 1 though phase 3 clinical trials, COVID-19 vaccines were also authorized for use in this population, with vaccine safety and immunogenicity assessed thereafter [18–23]. Early safety data for vaccination of SOTRs with 2 doses of mRNA-1273 or BNT162b2 were congruent with initial trials: adverse events were predominantly mild, local reactions; systemic reactions (eg, fever) were uncommon; and there was no clear evidence of increased incidence of postvaccination transplant rejection [24]. The introduction of vaccines against COVID-19 has reduced risks of severe disease and death in both immunocompetent and SOTR populations [13, 25]. Specifically, mortality decreased by 33% among SOTRs who had received 2 doses of an mRNA vaccine or 1 dose of the viral vector Ad26.COV2.S vaccine compared with SOTRs with no record of vaccination [13]. Nevertheless, rates of breakthrough COVID-19 infection and death remained higher among SOTR regardless of organ types compared with nontransplant controls [12].

The high risk of COVID-19–related morbidity and mortality faced by SOTRs necessitates a thorough understanding of vaccine benefit in this vulnerable population to better inform and advance COVID-19–related clinical practice. In this article, the immunogenicity, efficacy, and real-world effectiveness of COVID-19 vaccination in SOTRs, as well as the effect of immunosuppressive therapies on these outcomes, will be reviewed with respect to the 2 US Food and Drug Administration–approved mRNA-based COVID-19 vaccines, mRNA-1273 (SPIKEVAX; Moderna, Inc., Cambridge, MA, USA) and BNT126b2 (COMIRNATY; Pfizer, Inc., New York, NY, USA; BioNTech Manufacturing GmbH, Mainz, Germany), as well as different strategies for boosting immune responses in SOTRs. Finally, the current consensus guidelines for COVID-19 vaccination in SOTRs are presented alongside an evaluation of the challenges associated with implementing these recommendations into clinical practice.

## RESPONSES TO THE FIRST AND SECOND COVID-19 VACCINE DOSES AMONG SOLID ORGAN TRANSPLANT RECIPIENTS

During the early stages of the COVID-19 pandemic, immunocompromised individuals, including SOTRs, were identified as highly susceptible to severe outcomes and death; this population was thus prioritized for COVID-19 vaccination [26–30]. The recommended COVID-19 vaccination schedule for SOTRs was initially similar to that for immunocompetent adults: (1) 2 doses of mRNA-1273 or BNT162b2, with timing of 4 or 3 weeks between administrations, respectively; or (2) a single dose of Ad26.COV2.S vaccine [31]. Early immunogenicity data indicated that only 15% of SOTRs mounted appreciable antibody responses to the first dose of a COVID-19 mRNA vaccine [32, 33], with 39% of the nonresponders producing antibodies after the second dose. No antibody responses were detected after 2 vaccine doses among the remaining 46% of SOTRs [33]. Such suboptimal immunogenicity, while not unexpected, contrasts with the robust immune responses elicited by 2 doses of an mRNA vaccine among immunocompetent individuals, including seroconversion of 100% of healthy participants in most studies [18, 20]. Larger studies have subsequently confirmed and expanded upon findings of suboptimal immune responses to COVID-19 vaccination among SOTRs (Table 1 [32–62]). While pooled seroconversion rates of <58% have been reported for all SOTRs after 2 vaccine doses, rates vary by organ transplant type and are considerably heterogenous across studies [37, 38, 63, 64]. For example, among liver transplant recipients, seroconversion rates ranging from 38% to 80% have been reported [23, 34, 64]. However, seroconversion rates are lower among kidney (17%–75%), heart (18%–62%), and lung (18%–49%) transplant recipients [34, 41, 42, 44, 45].

**Table 1.** Vaccine Responses in Recipients of Solid Organ Transplants

Publication	Transplant Type	Vaccine(s)	Participants With Immune Responses, %^a^	Other Outcome(s) Measured^b^
**Second dose**				
Boyarsky et al, JAMA; 2021 [33]	SOTRs	mRNA-1273, BNT162b2	Humoral: 54%	Humoral response: 43% and 82% of SOTRs with and without antimetabolite therapy, respectively
Mazzola et al, Clin Infect Dis; 2022 [34]	SOTRs	BNT162b2	Humoral: 29% SOTRs; 38% LTRs; 35% HTRs; 17% KTRs	Age, triple IS therapy, and diabetes were associated with nonresponse
Reischig et al, Am J Transplant; 2022 [35]	KTRs	BNT162b2	Humoral: 16%; cellular: 71%	Breakthrough infection and mortality rates: 16% and 9%, respectively (22% and 14% in unvaccinated KTRs)
Hall et al, Am J Transplant; 2021 [36]	SOTRs	mRNA-1273	Humoral: 35%; cellular: 48%	NR
Benotmane et al, JAMA; 2021 [54]	KTRs	mRNA-1273	Humoral: 40%	NR
Sanders et al, Transplantation; 2022 [37]	KTRs	mRNA-1273	Humoral: 57%; cellular: 16%	Age, MMF/MPA therapy, and not using steroids was significantly associated with nonresponse after D2
Cucchiari et al, Am J Transplant; 2021 [38]	KTRs	mRNA-1273	Cellular or humoral: 65%; humoral: 30%; cellular: 35%	NR
Al Jurdi et al, Kidney Int; 2022 [39]	KTRs	mRNA-1273, BNT162b2	Humoral, nAb: wild type (29%), delta (24%), omicron (0%)	NR
Debska-Slizien et al, Vaccines; 2021 [40]	KTRs	mRNA-1273, BNT162b2	Humoral: 51%	Age and receipt of >2 IS therapies significantly associated with lower antibody titers
Speich et al, Clin Infect Dis; 2022 [41]	KTRs, lung TRs	mRNA-1273, BNT162b2	Humoral: 75% KTRs; 49% lung TRs	Seroconversion rates were higher among SOTRs receiving dual IS (86%) than those receiving ≥3 IS (54%) therapies
Shostack et al, Lancet; 2021 [42]	Lung TRs	BNT162b2	Humoral: 18%	Older age and the receipt of antimetabolite or mTOR inhibitors as part of IS therapy were associated with a lower likelihood of developing a humoral immune response
Hirama et al, J Infect Chemother; 2022 [43]	Lung TRs	mRNA-1273, BNT162b2	Humoral: 24%	Nonresponse was associated with increasing age and use of MMF at high concentrations
Hallet et al, J Heart Lung Transplant; 2021 [44]	HTRs, lung TRs	mRNA-1273, BNT162b2	Humoral: 62% SOTRs; 62% HTRs; 36% lung TRs	Antimetabolite IS therapy and <6 y since transplantation were associated with lower likelihood of antibody response development
Peled et al, J Heart Lung Transplant; 2021 [45]	HTRs	BNT162b2	Humoral: 18%	NR
Herrera et al, Am J Transplant; 2021 [46]	LTRs, HTRs	mRNA-1273	Humoral: 71% of LTRs, 57% of HTRs; cellular: 86% of LTRs, 70% HTRs	Age, MMF use, vaccination <1 y after transplant, and hypogammaglobulinemia were predictors of poor immune responses
**Third dose**				
Werbel et al, Ann Int Med; 2021 [47]	SOTRs	mRNA-1273, BNT162b2, Ad26.COV2.S	Humoral: 47%	NR
Kumar et al, Am J Transplant; 2022 [48]	SOTRs	mRNA-1273	Humoral, nAb: 70%; omicron-specific: 18%	Age, transplant type, or IS therapy were not associated with omicron-specific nAb at 1 mo after D3
Kamar et al, NEJM; 2021 [49]	SOTRs	BNT162b2	Humoral: 68%; 44% of nonresponders after D2	NR
Hall et al, NEJM; 2021 [50]	SOTRs	mRNA-1273	Humoral, nAb: 60%	NR
Tylicki et al, Vaccines; 2021 [51]	KTRs	mRNA-1273, BNT162b2	Humoral: 97% of previously infected KTRs; 71% of immune-naive KTRs (47% of nonresponders after D2)	NR
Reindl-Schwaighofer et al, JAMA Intern Med 2022 [52]	KTRs	mRNA-1273, BNT162b2, Ad26.COV2.S	Humoral: 39%; nAb: 22%	IS type and time elapsed since last kidney transplant were significantly associated with development of an antibody response
Odriozola et al, Transplantation; 2022 [53]	LTRs	mRNA-1273	Humoral: 97%; seroconversion in 75% of D2 nonresponders	NR
Benotmane et al, JAMA; 2021 [54]	KTRs	mRNA-1273	Humoral: 49%; 27% of previous nonresponder KTRs; 81% with weak responses after D2	NR
Charmetant et al, Sci Transl Med; 2022 [55]	KTRs	mRNA-1273, BNT162b2	Humoral, nAb: 39%–41% of nonresponders after D2	NR
Al Jurdi et al, Kidney Int; 2022 [39]	KTRs	mRNA-1273, BNT162b2	Humoral, nAb: 61% (wild type); 59% (delta); 12% (omicron)	Breakthrough infection: 6% after D3
Stumpf et al, Front Med; 2022 [56]	KTRs	mRNA-1273, BNT162b2	Humoral: 19%–49%; cellular: 6%–38%	Seroconversion rates with heterologous vaccination: 50% mRNA-1273 (D1 and D2)/BNT162b2 (D3); 36% BNT162b2 (D1 and D2)/mRNA-1273 (D3)
**Fourth dose**				
Alejo et al, Transplantation; 2021 [57]	SOTRs	mRNA-1273, BNT162b2	Humoral: 83% with high titers; 50% of nonresponders after D3	MMF, tacrolimus, and corticosteroid use were associated with nonresponse after D4
Masset et al, Kidney Int; 2022 [58]	KTRs	BNT162b2	Humoral: 43% of nonresponders after D3	Breakthrough infection: 1/49 (2%) Longer time between D3 and D4 and lower steroid use was noted among D4 responders
Thomson et al, EClinicalMedicine; 2022 [59]	KTRs	mRNA-1273, BNT162b2, ChAdOx1	Humoral: 81% of infection-naive KTRs; 25% of nonresponders after D3	Shorter intervals between D3 and D4 were associated with a greater likelihood of seroconversion
Midtvedt et al, Am J Transplant; 2022 [60]	KTRs	mRNA-1273, BNT162b2	Humoral: 42% of lowor nonresponders after D3; 28% of nonresponders	Antibody levels and renal function at the time of D4 receipt are associated with response
Benotmane et al, Kidney Int; 2022 [61]	KTRs	mRNA-1273	Humoral: 81% of poor responders after D3 developed strong response; 66% of KTRs with delta-specific nAb (16% before D4)	Previous nonresponse and triple IS therapy (tacrolimus, MMF, and steroids) were associated with lower neutralizing capacity
Tylicki et al, Arch Med Sci; 2022 [62]	KTRs	mRNA-1273	Humoral: 42% of nonresponders after BNT162b2 D3	NR

Abbreviations: D, dose; HTR, heart transplant recipient; IS, immunosuppression; KTR, kidney transplant recipient; LTR, liver transplant recipient; MMF, mycophenolate mofetil; MPA, mycophenolate acid; nAb, neutralizing antibodies; NR, none reported; SOTR, solid organ transplant recipient; TR, transplant recipient.

Includes any detectable SARS-CoV-2–specific humoral or cellular responses.

Includes seroresponse rates stratified by IS therapy type and heterologous vaccination, predictors of immune response to vaccination, breakthrough infection rates, and mortality.

Few studies have addressed cellular immune responses in SOTRs after 2 doses of a COVID-19 vaccine, and results are variable, partly owing to the application of different assays [37, 38, 46]. Measurement of T-cell responses by interferon (IFN)-γ release assay demonstrated that compared with the 84% of nontransplant controls that develop high T-cell responses after 2 vaccine doses, the small proportion of SOTRs (16%) with T-cell responses had significantly lower IFN-γ production [37]. Data generated by IFN-γ ELISpot assay and stratified by transplanted organ type revealed that cellular immune responses were elicited among 33% to 86% of lung, 70% of heart, and 30% of kidney transplant recipients that had no detectable humoral responses after 2 doses of the mRNA-1273 vaccine [36–38, 46, 65]. Assessment of polyfunctional IFN-γ and interleukin-2–expressing T cells indicated the induction of a cellular immune response in 48% of SOTRs after 2 doses of mRNA-1273 [36]. While a strong, multifaceted immune response has been associated with a reduced severity of SARS-CoV-2 infection in general, the presence of cellular immune responses may be hypothesized to partially compensate for a lack of neutralizing antibodies in SOTRs [66]. Nevertheless, the functional relevance of individual facets of the immune system for protection against infection and severe COVID-19–related outcomes in SOTRs remains to be elucidated.

Overall, transplant status has been independently associated with an increased risk for breakthrough infections after 2 vaccine doses (24%) relative to immunocompetent individuals (21%) [13]. Among SOTRs, recipients of heart transplants had the greatest incidence of breakthrough infections after 2 vaccine doses, while lung transplant recipients were at higher risk for COVID-19–related hospitalization and death [13]. US kidney transplant recipients had a breakthrough infection rate of 16% after 2 vaccines doses compared with a 22% infection rate in unvaccinated controls [35]. Corresponding to this, a reduction from 13% to 8% in case-fatality ratio was reported in the United Kingdom for SOTRs vaccinated with 2 doses of either ChAdOx1-S (VAXZEVRIA; AstraZeneca, Cambridge, UK; Oxford University, Oxford, UK) or BNT162b2 [67].

COVID-19 vaccination offers significant benefits with respect to severe COVID-19–related outcomes in SOTRs, including lower risk of mechanical ventilation and mortality [13, 68, 69]. However, SOTRs who are vaccinated with 2 doses still have an 82-fold increased risk of breakthrough infection and a 10- to 485-fold higher risk of mortality than the general immunocompetent population [13, 70]. Therefore, SOTRs remain at high risk of inadequate immune responses and poor COVID-19–related outcomes; such factors warranted the recommendation of a third vaccine dose for SOTRs.

## IMMUNE RESPONSES TO A THIRD COVID-19 VACCINE DOSE IN SOLID ORGAN TRANSPLANT RECIPIENTS

The suboptimal immune responses observed for SOTRs who received a typical 2-dose schedule of a COVID-19 vaccine prompted public health authorities to recommend the incorporation of additional vaccine doses for this patient population [31, 71]. Receipt of a third dose of mRNA-1273 (full 100-µg dose) or BNT162b2 (≥12 years of age, 30-µg dose; 5–11 years of age, 10-µg dose) is now recommended for SOTRs ≥4 weeks after a second dose of an mRNA vaccine or ≥2 months after 2 doses of the recombinant protein vaccine, NVX-CoV2327 (COVOVAX; Novavax, Inc., Gaithersburg, MD, USA; Serum Institute, Pune, India) [31]. For SOTRs vaccinated with 1 dose of Ad26.COVS.2, an additional dose of either of the mRNA vaccines is also recommended 4 weeks following vaccination to boost immune responses [31]. Following the additional dose of Ad.26.COV2 or the third mRNA vaccine dose, seroconversion rates of 39% to 97% were achieved among SOTRs [47, 49, 51–53]; 33% to 49% of nonresponders who were seronegative after 2 doses developed immune responses, and antibody levels were boosted in >80% of previously seroconverted SOTRs (Table 1 [32–62]) [47, 49, 54, 72]. As the presence of neutralizing antibodies may be correlated with protection against COVID-19, the lower proportion of SOTRs with these neutralizing antibodies against wild-type SARS-CoV-2 (21%–61%) even after a third vaccine dose remains a concern [39, 52, 55, 73].

Although there is still room for improved immune responses to COVID-19 vaccination in a proportion of SOTRs vaccinated with 3 doses, the third dose significantly reduced the risk of SARS-CoV-2 infection (8% vs 26% in unvaccinated), hospitalization (3% vs 10%), and death (<1% vs 8%) among SOTRs [74], demonstrating a substantial benefit for SOTRs receiving a third dose. Factors associated with nonresponse to a third dose of a COVID-19 vaccine in this population included type of vaccine received for dose 1 and dose 2, immunosuppressive therapy type, and the time elapsed since transplant. In contrast to the first 2 vaccine doses, immune responses to a third dose do not appear to be predicated upon age, time elapsed since the previous vaccine dose, or the number of organ transplants received [52].

## ADDITIONAL APPROACHES FOR ENHANCING IMMUNE RESPONSES AND COVID-19–RELATED OUTCOMES AMONG SOLID ORGAN TRANSPLANT RECIPIENTS

The emergence of SARS-CoV-2 variants brought about new concerns regarding the evasion of vaccine- and infection-acquired immune responses by these variants. Although the beta and delta variants exhibited moderate levels of immune evasion, COVID-19 vaccine effectiveness remained largely intact in immunocompetent individuals during the periods of beta and alpha dominance [75]. Among SOTRs, a similar proportion of individuals developed at least some degree of neutralization against wild-type SARS-CoV-2 (29%–78%) and the delta variant (24%–75%) after 2 vaccine doses [39, 76], which may suggest congruence with immunocompetent individuals regarding vaccine effectiveness, although this remains to be determined.

By contrast, the omicron variant is associated with increased infection and severe disease among immunocompetent individuals vaccinated with 2 doses, and its emergence necessitated the receipt of additional doses to boost immune responses [75]. Naturally, the omicron variant is also a major concern for immunocompromised populations. Following 2 vaccine doses, omicron-specific neutralizing antibodies were completely absent among SOTRs. However, a third dose resulted in the development of omicron-specific neutralizing antibodies among 12% to 25% of SOTRs [39, 73, 76], demonstrating the importance of following the revised vaccine recommendations. In a cohort of SOTRs that experienced breakthrough infection (6%) after 3 vaccine doses, none had omicron-specific neutralizing antibodies [39], supporting the use of additional vaccine doses in SOTRs to enhance protection. In addition to SARS-CoV-2 variant emergence, waning immune responses are a major factor underlying the rationale for recommending additional doses. Among seroconverted kidney transplant recipients, < 60% remained positive for neutralizing and binding antibodies 6 months after 2 vaccine doses and only 37% exhibited cellular responses, compared with 79% of nontransplant controls [77].

In light of these concerns, a third, if Ad26.COV2.S was administered as dose 1, or fourth COVID-19 vaccine dose was recommended for this vulnerable population [31]. Among SOTRs vaccinated with mRNA-1273 or BNT126b2, a fourth dose of either of these approved mRNA vaccines should be scheduled ≥2 months after the third. For SOTRs who received Ad26.COVS.2, receipt of an mRNA vaccine ≥2 months after Ad26.COV.2 is recommended [31]. Seroconversion was obtained among a marginal number (25%–50%) of kidney transplant recipients who were previous nonresponders (Table 1 [32–62]) [57–60], warranting the investigation of protective alternatives for SOTRs who are nonresponders to a third dose. The number of transplants received, the type of immunosuppression used, and the time elapsed between the third and fourth dose were associated with a lack of seroconversion [58, 59]. By contrast, a fourth dose of a COVID-19 vaccine enhanced antibody titers among low responders (81%–100%), as well as those with high antibody levels after dose 3 [57, 59–61]. Additionally, the proportion of SOTRs with delta strain–specific neutralizing antibodies increased from 16% to 66% after the fourth vaccine dose [61]. Regarding cellular immune response, positive anti-spike antibody levels and time elapsed since transplant are strong predictors of the induction of spike-specific T-cell responses in SOTRs [78]. Together, these results demonstrate substantial benefit of additional doses among SOTRs with responses to previous vaccine doses.

Another approach to enhancing immune responses among SOTRs is the use of heterologous vaccination schedules, involving “mixing and matching” of vaccines from different platforms (eg, Ad26.COV2.S or the inactivated virus CoronaVac [Sinovac Biotech, Beijing, China] with BNT162b2), or different vaccines from the same platform (eg, mRNA-1273 and BNT162b2). Heterologous vaccination has been implemented to safely enhance vaccine coverage and protection against severe COVID-19 disease in the general population [79–81] and is thus a potential strategy to improve immune responses among SOTRs. However, data are variable regarding the benefits of heterologous vaccination for SOTRs.

Seroconversion rates following a third vaccine dose were significantly higher among SOTRs vaccinated with a homologous BNT162b2 schedule than those who first received ChAdOx1 [59]. Nevertheless, neutralizing antibody titers increased more than 4-fold following a heterologous third dose of an mRNA vaccine in SOTRs who received ChAdOx1 [82], indicating such heterologous dosing may be substantially beneficial in cases where mRNA vaccines are limited. A fourth dose of BNT162b2 among SOTRs who received homologous BNT126b2 (doses 1–3) and heterologous ChadOx1 (doses 1 and 2)/BNT126b2 (dose 3) vaccinations resulted in similar proportions of seropositive SOTRs (86% and 82%, respectively) [59], supporting the benefit of introducing heterologous vaccines at the third dose.

Results of heterologous dosing with a third dose of Ad26.COV2.S were not significantly better in terms of antibody development 4 weeks postvaccination than homologous mRNA vaccination in SOTRs who were nonresponders [52, 83]. Interestingly, by 6 months postvaccination, heterologous vaccination resulted in significantly higher titers and a greater seroconversion rate among SOTRs (80% vs 59% for homologous) [83]. Congruently, vaccination with heterologous regimens consisting of an mRNA vaccine and ≥1 dose of a viral vector vaccine has been associated with a positive spike-specific T-cell response among SOTRs [78]. This suggests differential long-term immunogenicity between platforms and is consistent with the gradual increase in immune responses following Ad26.COV2.S vaccination among the general population [84, 85]. While not statistically significant, humoral immune responses were higher among SOTRs receiving a third dose of an mRNA vaccine if their first dose had also been an mRNA vaccination rather than CoronaVac [86]. A similar trend was observed among patients with end-stage renal disease [87], suggesting that in cases where mRNA vaccines and CoronaVac are available as third doses, SOTRs and other immunocompromised populations could benefit from homologous mRNA vaccination schedules.

In addition to cross-platform vaccination schedules, heterologous schedules consisting of vaccines from the same platform have been studied [62, 88]. Vaccination with mRNA-1273 among nonresponders who received 3 doses of BNT162b2 resulted in seroconversion of almost half of previous nonresponder SOTRs and led to higher titers than in SOTRs vaccinated with 3 homologous mRNA vaccine doses [62, 88]. These data suggest that combining vaccines from the same platform may offer some benefit in cases where classical heterologous boosting with other vaccine platforms is limited. While more data are required to confirm these findings, these studies demonstrate that immune responses among SOTRs are predicated on the type of vaccine used. Another important predictor of immunogenicity among SOTRs following COVID-19 vaccination is the type of immunosuppressive therapy used; the effect of these therapies on responses will be discussed next.

## THE INFLUENCE OF IMMUNOSUPPRESSIVE THERAPIES ON IMMUNE RESPONSES TO COVID-19 VACCINATION IN SOLID ORGAN TRANSPLANT RECIPIENTS

Studies reporting the effect of corticosteroids on immune responses to COVID-19 vaccination among SOTRs are variable. Corticosteroid use among SOTRs has been associated with greater seroconversion rates after 2 doses of an mRNA vaccination [37]; however, low or absent neutralizing antibody responses have also been reported [89]. The use of induction therapies, including alemtuzumab, anti-thymocyte globulin, basiliximab, and rituximab, are typically associated with low antibody responses and seroconversion rates after COVID-19 vaccination [90–93]. Induction therapy use may, at least in part, be responsible for the lower vaccine responses observed among SOTRs with recent transplants than those with less recent transplants [37, 52, 93]. Congruent with this, the time elapsed since rituximab treatment has been reported as a predictor of immune responses to 2 mRNA COVID-19 vaccine doses [92]. Regarding maintenance regimens, azathioprine and calcineurin inhibitors are associated with poor antibody responses and neutralization, respectively, among SOTRs after 2 doses of an mRNA vaccine [37, 89]. Belatacept, commonly used to maintain immunosuppression in kidney transplant recipients, is a significant predictor of low seroconversion among SOTRs, even after 3 doses of an mRNA vaccine [94–96]. Among SOTRs who received non–mRNA-based COVID-19 vaccines, belatacept is also associated with low seroconversion rates [97]. Irrespective of dose number, MMF/mycophenolate acid use, particularly in high doses and within triple immunosuppressive therapy, is strongly associated with low seroconversion rates and weak immunologic, but not cellular, responses to COVID-19 vaccination among SOTRs [32, 37, 46, 78]. Interestingly, MMF treatment predicts poor humoral responses to 2 doses of a vaccine in SOTRs who are COVID-19 infection-naive; however, this treatment is not associated with dampened immune responses among those who have recovered from COVID-19 [98]. Everolimus is a promising alternative to MMF use for preventing organ rejection; after 3 vaccine doses, a seroresponse rate of 100% and significantly higher antibody levels were observed among SOTRs treated with everolimus (38% for MMF) compared with SOTRs treated with MMF. Everolimus use was not associated with improved cellular immune responses after 3 doses [99].

## THE DYNAMIC LANDSCAPE OF COVID-19 VACCINATION GUIDELINES AND CLINICAL PRACTICE FOR SOLID ORGAN TRANSPLANT RECIPIENTS

As the COVID-19 pandemic progressed and vaccines were developed and authorized for emergency use, guidelines for vaccine deployment were published. Although SOTRs were excluded from the initial clinical trials, COVID-19 vaccines were authorized for emergency use in this population. The subsequent study of vaccine safety and immunogenicity revealed that SOTRs required more doses than immunocompetent individuals, necessitating guideline revisions to ensure optimal protection of this vulnerable population [71, 100–103]. The COVID-19 treatment and vaccination guideline landscape for SOTRs is therefore continuously evolving, influenced by the discovery of new information, professional disease and transplant society guidelines, physician and patient perception, as well as the dynamic nature of the COVID-19 pandemic itself (Table 2). Importantly, guidelines strongly encourage pretransplant patients and SOTRs to adhere to vaccination schedules, given their increased risk for severe disease and death due to COVID-19 [71, 101, 103–105]. These guidelines are based on an array of evidence documenting vaccine efficacy and safety [101, 103–105].

**Table 2.** Summary of Key Considerations for COVID-19 Vaccination in Solid Organ Transplant Recipients

Additional vaccine doses	Given that the recommended additional COVID-19 vaccine doses enhance protection in SOTRs overall, the timely receipt of vaccines according to current guidelines will be crucial
Vaccine type and regimen	The use of heterologous and/or cross-platform vaccination regimens where possible could ensure the achievement of optimal outcomes for SOTRs
Immunosuppressive therapies	Where multiple induction and/or maintenance immunosuppressant options are available, it will be important to select therapies that will minimize organ rejection risk without compromising vaccine responses
Vaccination guidelines	As more real-world evidence emerges and is incorporated into guidelines and clinical practice, cross-organization and international cooperation will mediate the broadest possible uptake and application of guidelines

Abbreviation: SOTRs, solid organ transplant recipients.

**Figure 1.**
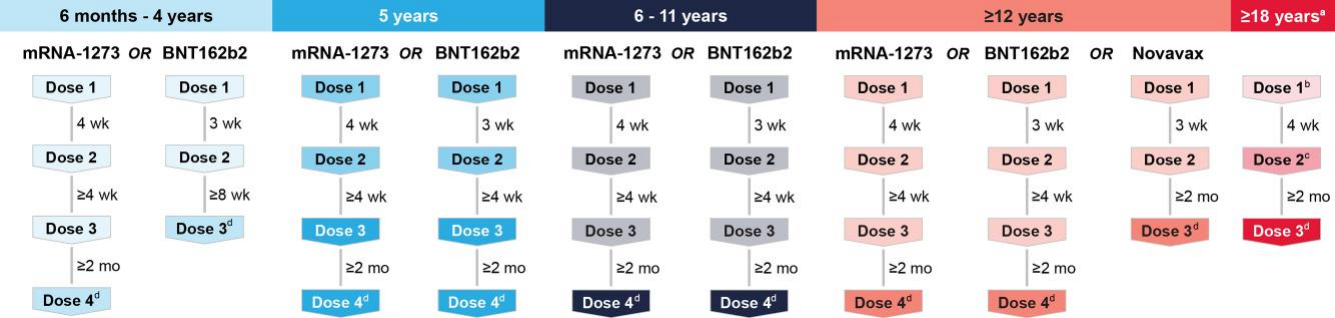
Clinical guidelines for COVID-19 vaccination in solid organ transplant recipients. Recommended COVID-19 vaccination schedules by age group. ^a^SOTRs who received Ad26.COV2.S as dose 1; ^b^Ad26.COV2.S; ^c^mRNA vaccine; ^d^Bivalent mRNA vaccine. Abbreviation: SOTRs, solid organ transplant recipients.

Current guidelines provide optimal timing between doses and recommendations for vaccination of pretransplant patients and SOTRs receiving immunosuppressive therapies. The Centers for Disease Control and Prevention, American Society of Transplantation, American Society of Transplant Surgeons, and the Advisory Committee on Immunization Practices recommend all SOTRs stay up-to-date with recommended COVID-19 vaccines for their age group [71, 103–105] (Figure 1). Except for those vaccinated with Ad26.COV2.S and those aged 6 months to 4 years receiving BNT162b2, it is recommended that SOTRs receive 3 doses of mRNA-1273 or BNT162b2 separated by 3 or 4 weeks (vaccine and age group dependent; Figure 1), followed by a bivalent mRNA vaccine as a fourth dose ≥2 months following the third dose [103]. A bivalent mRNA vaccine is recommended ≥2 months after the completion of a 2-dose NVX-CoV2327 in SOTRs aged ≥12 years. Adult SOTRs vaccinated with the 1-dose series of Ad26.COV2.S should receive an additional mRNA vaccine dose 4 weeks later and a bivalent mRNA vaccine ≥2 months thereafter [103]. Routine testing to determine antibody responses after vaccination is not recommended on the basis that commercially available tests do not measure cellular responses, are not all quantitative, and are not predictive of infection risk [24, 103, 105]. COVID-19 vaccination should not be delayed among SOTRs receiving immunosuppressive therapy; however, SOTRs should be evaluated on a case-by case basis to consider current and planned immunosuppressive therapy to optimize the patient's condition and expected response to vaccination, as well as individual benefits and risks [103]. When possible, COVID-19 vaccination should be administered ≥2 weeks before the initiation or resumption of therapies, yet professional organ transplant societies recommend against adjusting therapies for the purposes of vaccination [71, 103, 105]. Anti-SARS-CoV-2 monoclonal antibodies (eg, Evusheld) are currently obsolete and not authorized for prophylactic use due to the resistance of the predominant circulating omicron subvariants [106–108]. Nevertheless, should next-generation monoclonal antibodies products emerge, these will likely be important to expand protection of vulnerable immunocompromised populations, including SOTRs.

While guideline revisions favor the implementation of new evidence into patient care, such rapidly evolving ideas may hinder uptake and application of recommendations in clinical practice. The generation of real-world evidence that support existing COVID-19 vaccination guidelines for SOTRs will be crucial to enhance care delivery for this vulnerable population.

## CONCLUDING REMARKS

SOTRs are at high risk for morbidity and mortality due to infections, including SARS-CoV-2. The use of certain types of immunosuppressive therapies that prevent organ rejection is generally associated with poor immune responses to vaccination. As expected, responses to 2 doses of a COVID-19 vaccine in SOTRs are typically poor; however, responses are largely improved following a third dose. While more data are needed to confirm initial findings, heterologous vaccination schedules may also offer promise for SOTRs who have low or absent responses after 2 or 3 vaccine doses. A fourth dose has also proved beneficial for further enhancement of immune responses overall as well as generating omicron variant–specific neutralizing antibodies in some SOTRs. Nevertheless, a proportion of SOTRs remain nonresponsive, even after a fourth dose, warranting the investigation of alternative measures to protect these vulnerable individuals. Another unanswered question is the longevity of vaccine protection and whether SOTRs would require annual immunization against newer SARS-CoV-2 variants. Although SOTRs were excluded from initial clinical trials, subsequent assessments have documented excellent safety profiles for COVID-19 vaccines in this group, and no risk of transplant organ rejection has been identified. A comprehensive understanding of COVID-19 vaccine immunogenicity, efficacy, and real-world effectiveness is crucial for informing public health strategies and updating the ever-evolving vaccination guidelines to help protect vulnerable immunocompromised populations, including SOTRs.

## Notes

***Acknowledgments.*** Medical writing and editorial assistance were provided by Jessica Nepomuceno, PhD, of MEDiSTRAVA in accordance with Good Publication Practice (GPP 2022) guidelines, funded by Moderna, Inc., and under the direction of the author.

***Supplement sponsorship***. This article appears as part of the supplement “COVID-19 Vaccination in the Immunocompromised Population,” sponsored by Moderna, Inc.
